# Syndemics, Infodemics, and How the Severity of COVID-19 Was Aggravated by Our Maladapted Lifestyle and by the Political Handling of Public Health

**DOI:** 10.3389/fpubh.2021.763645

**Published:** 2021-09-09

**Authors:** Jeff M. P. Holly

**Affiliations:** Faculty of Medicine, School of Translational Health Science, Bristol Medical School, University of Bristol, Southmead Hospital, Bristol, United Kingdom

**Keywords:** metabolism, public health, diabetes, obesity, COVID-19

The current COVID-19 pandemic has had devastating consequences across the globe, causing the greatest public health crisis in over a century. To address this, global science has been mobilized as never before resulting in an infodemic of publications that has been made publicly available in the COVID-19 open Research Dataset (CORD-19). This depository of information rapidly grew beyond the size that any human could possibly read. In their recent paper (1), the authors have taken a novel approach to address both the infodemic and also the most pressing issue of the pandemic: why the majority of cases experience mild to moderate symptoms, but a few develop severe life-threatening disease. Employing machine reading and knowledge engineering tools they managed to extract common linked threads from the vast literature and condense these into a cohesive hypothesis. Their conclusion was that perturbed glucose metabolism was the most prevalent underlying factor predisposing individuals to a more severe COVID-19. There has been robust consensus that age, diabetes mellitus (DM), obesity and hypertension are all strong risk factors for developing severe COVID-19 and disturbed glucose metabolism is common to all these conditions.

Throughout evolution species have adapted to best survive in their prevailing environment. In the context of the current pandemic, genetic analysis has revealed that three of the genetic variants strongly linked to severity of COVID-19 entered the human population by gene flow from Neanderthals or Denisovans some 40,000–60,000 years ago (2, 3). Neanderthals in Eurasia and Denisovans in the Asia-Pacific region evolved throughout the ice-age and sought shelter in caves which they presumably cohabited with bats and both of these archaic human species acquired genetic variants that provided a survival advantage to the bat RNA-viruses to which they were exposed. The acquisition of these variants then also helped to facilitate genetic adaptation of early humans to viral challenges (4). On a more fundamental level, from the beginning it was always essential that the activity, and hence metabolism, of any organism had to be tightly adapted to the supply of nutrients and metabolic fuels and this adaptation was originally acquired by key enzymes and their regulators being directly responsive to levels of nutrients. Hence in single cell organisms, such as yeast, the evolutionary conserved, pivotal intracellular regulator of metabolic pathways, phosphatidylinositol-3 kinase (PI3K), is directly activated by the availability of amino acids (5). With the evolution of metazoa it became important that all cells in multi-cellular organisms were coordinately regulated. This adaptation was enabled by PI3K no longer being a direct nutrient sensor but evolving into a heterodimer with a catalytic subunit and a regulatory subunit, with the latter activated by cell surface receptors that could respond to social signals, classically the insulin receptor (IR), to ensure the synchronous control of metabolism within all cells that constitute the organism (6). Over most of human evolution nutrients were in limited supply and adaptations that provided an advantage were the “thrifty genes” that enabled efficient storage of energy to sustain activity between intermittent meals. In mammals this occurs within fat stores that can then be mobilized, to ensure optimal energy supplies for cell metabolism and homeostasis of systemic glucose levels, over periods of prolonged fasting. However, over the very recent past human lifestyles have rapidly changed due to the unlimited availability of highly-processed, energy-dense foods that resulted, for the first time throughout their evolution, in humans living in a post-prandial state for much of the day. As a consequence of this sudden change the evolved thrifty genotype was no longer an advantage but now was a maladaptation resulting in excessive accumulation of fat, which in turn activated feedback mechanisms, to reduce further glucose uptake, leading to insulin resistance and eventually diabetes. The clinical sequalae of this has been the gradual spreading, global pandemics of obesity, DM and cardiovascular disease that threaten to overburden health services and with which we are all familiar. Over recent years it has also become apparent that many of the common cancers can be added to this list of sequalae, as surveys of public health trends indicate that, with the decline in tobacco usage, metabolic dysregulation will soon overtake smoking as the leading modifiable cause of many cancers across North America and Europe (7, 8). If these were not already sufficient reasons for this pandemic of disturbed metabolic health to be a major issue facing Public Health, the new evidence synthesis presented by Logette et al. (1) elegantly details how compromised metabolism has additionally ill-prepared us to cope with a global viral pandemic. From their synthesis of the vast CORD-database they nicely outline how disturbed glucose metabolism lowers the many defenses within the body that protect us from respiratory viruses. This appears to be particularly true for zoonotic viruses that cross-over from species that originally shared homes with our distant ancestors in caves. The coherent story generated from the machine learning synthesis raises many interesting issues: including how we handle the current COVID-19 pandemic, our preparedness for future pandemics, the capabilities of Public Health to cope with such a crisis and the delivery of critical care when services are stretched to the limit. From their synthesis the authors suggest that specific treatments widely used to treat metabolic abnormalities, such as metformin and a ketone diet, should be examined in trials to ameliorate the severity of COVID-19. Managing glucose homeostasis in severely ill patients is, however, challenging at the best of times (9) and these suggestions raise important questions regarding critical care management during a pandemic. The dramatic spread of COVID-19 rapidly overwhelmed existing ICU capacity in many parts of the world with an exponential increase in the number of patients requiring multi-organ support. Critically the supply of specialist intensive care nurses and doctors was rapidly out-stripped, and this was exacerbated by staff themselves becoming infected. This resulted in the conscription of staff from other specialties, many without prior training in critical care or airway management. As a consequence, there have been many recent recommendations for preparing ICUs to deal with such a crisis. These include employing trained intensive care doctors in a role supervising the delivery of care by non-specialist staff (10, 11). One suggestion has been to break-down intensive care into its constituent parts and create an “assembly line” approach focused on standardization, simplification, upskilling and multidisciplinary teamworking (12). In future such teams should include specialists with expertise in metabolic management.

The current pandemic has accelerated the infodemic and in many fields it has already become almost impossible to keep abreast of the volume of new publications. The approach taken by Logette et al. has started the development of tools that will clearly be invaluable in the future for interpretation of the ever-increasing information flow. They nicely describe both the strengths of their approach and some of the issues that will still need to be addressed to ensure that such tools will generate an objective distillation of the literature into a synopsis that can be humanly assimilated. Their distillation of the COVID-19 literature provides an interesting, rational story that raises many interesting questions both regarding our way forward in the current pandemic, how we should prepare for future pandemics and more fundamental questions regarding how Public Health should address future, even more daunting, challenges.

The synthesis of evidence from Logette et al. indicates what we can learn from the vast literature current pandemic. Their evidence adds further to the imperative for Public Health to address the growing pandemic of metabolic ill-health in order to ameliorate future syndemics as more people become maladapted to respond to respiratory infections. Throughout the slow pandemic of metabolic disorders it has, however, become clear that acquiring the scientific knowledge alone does not necessarily translate to this knowledge being implemented. For many decades clear paradoxes have existed that present obstacles to the implementation of what we have learned. We know that poor nutrition results in the metabolic disorders that are associated with obesity and we know the means to prevent or cure these disorders in most people (13). From this knowledge many governments have developed public health advice for their citizens, encouraging them to eat more fruit and vegetables and consume less meat and dairy. However, the same governments, at the same time, also provide substantial subsidies to farming and agriculture predominantly for them to produce meat and dairy but provide little, or no, support for the production of the fresh fruit and vegetables. An analysis estimated that 56.7% of dietary energy intake in the USA was derived from federal subsidized foods (14). The agricultural policy, with large farming subsidies, is thought to contribute significantly to the epidemics of obesity and poor metabolic health both in the USA (15) and in Europe (16). Farming subsidies were introduced a century ago after the first world war (and the Spanish influenza pandemic) when protein malnutrition was a large concern and these subsidies subsequently grew into major components of economies that are now sustained by powerful political lobbies. However, a century later nutritional needs have changed considerably with modern lifestyles and rather than helping to meet nutritional requirements these subsidies are now helping to fuel a pandemic of metabolic ill-health.

Very similar political obstacles have also become apparent during the current pandemic. The conclusions from Logette et al. indicate what we could learn from the current pandemic; to implement these lessons will require us to overcome these obstacles. In 2019, prior to the COVID-19 pandemic, the Johns Hopkins Center for Health Security, together with the Nuclear Threat Initiative (NTI) and the Economist Intelligence Unit (EIU) produced the Global Health Security Index (GHSI) an assessment of global health security capabilities in 195 countries (17). Based on the availability of expertise and resources the report ranked countries in their preparedness for a pandemic; the USA was ranked as most prepared with the UK ranked second. Soon into the current pandemic the shortcomings of this report became apparent as it did not account for political decisions: expertise and resources count for little if not appropriately implemented (18). Although an emerging pandemic was high on the risk registers of many countries, the funding and preparation for what many perceived as an inevitable risk lagged far behind that for other perceived risks such as terrorism or armed conflicts. Despite their GHSI rankings, national budgets for Public Health have generally not reflected any sense of preparedness for an emerging pandemic. In 2020 the USA devoted $750 billion of its federal budget to its military defense but only just over $0.5 billion for global health security (https://www.cfr.org/report/pandemic-preparedness-lessons-COVID-19/pdf/TFR_Pandemic_Preparedness.pdf). In the UK the national Public Health budget was reduced by 15% over the 6 years prior to 2019 (https://www.kingsfund.org.uk/projects/nhs-in-a-nutshell/spending-public-health); but then when the pandemic hit the UK, the equivalent of 10 years of the entire Public Health budget was spent on a test-and-trace system that had limited or no effect on the course of the pandemic (19). Thus, far in the COVID-19 pandemic the UK has already lost more than twice the civilian lives than were lost in the whole of World War II and the USA has lost more American lives than in all military conflicts since World War II (including those lost in Vietnam, Korea, Iraq, and Afghanistan). Knowing risks and learning how to deal with them will only be effective if this imbalance of political priorities is redressed.

The basic principles of Public Health management during an epidemic have been well-established for a long time and were proven to be effective in the recent viral epidemics such as those caused by Ebola, Zika, SARS, and MERS. During the current COVID-19 pandemic there have, however, been huge geographical variations in how these principles have been applied with consequent large variations in outcomes. Some countries such as South Korea, Taiwan, Vietnam, and New Zealand have been exemplars in applying good basic Public Health measures, and this has been accompanied by relatively low mortality rates. As of July 2021, recorded COVID-19 deaths per million of population were: South Korea 39; Taiwan 29; Vietnam 0.9; New Zealand 5. In contrast other countries have taken political decisions to either delay or only apply limited Public Health measures in order to protect their economies or to protect “civil liberties” and have considered that alternative approaches such as “herd-immunity” or a rapid development of vaccines may protect their populations. Politicians in countries such as the USA and UK tried these alternative strategies with dramatically different health outcomes, despite their presumed preparedness (COVID-19 deaths per million: USA 1,866; UK 1,879); in addition, these countries have also incurred much greater harm to their economies. An interesting recent comparison of the outcomes of the pandemic in terms of not just health, but also the impact on the economy and on restrictions of civil liberties; between five countries that adopted rigorous public health measures, in order to eliminate COVID-19, with 32 countries that took the alternative approach and attempted to mitigate the spread of COVID-19 and “balance” the damages, found marked differences in all three outcomes (20). The starkest difference was that the elimination strategy resulted in 25-times fewer deaths, but also the impact on their economies were much less and these countries returned faster to economic growth and in addition the restrictions of civil liberties were also far less, and of shorter duration, enabling citizens to regain their “freedoms” much sooner (20).

One of the cornerstones of a Public Health response to an outbreak of an infectious disease is to identify cases and then prevent the spread of infection by the application of testing, tracing contacts and their isolation to avoid further infections. This was proven to have been effectively applied in some of the poorest countries in the world during the 2014 Ebola outbreak in West Africa (21). During the current COVID-19 pandemic some countries, such as South Korea, Taiwan and Vietnam have very effectively applied test, trace and isolate by acting fast, before cases could overwhelm the system, and by employing local Public Health resources and modern technologies to identify cases and support and monitor their isolation (22). In contrast some of the most affluent nations, such as the USA and UK have failed to establish effective systems (22). In the UK a political decision was taken to not use existing public health resources but to establish a centralized system of case identification and contact tracing and centralized, newly-developed, laboratories; all of which were out-sourced to private companies with limited or no expertise either in pathology testing or implementing public health. As a consequence, there was delay in establishing a system resulting in high numbers of cases which the system then struggled to get to grips with. During the first wave of COVID-19 in the UK it was estimated that the test, trace and isolate system only managed to identify contacts of 8% of cases with symptoms (23). Given that there was also probably considerable transmission from pre-symptomatic or asymptomatic cases and very limited support, or monitoring, of contact isolation the system was widely considered to have had limited, or no, impact on the pandemic despite performing more tests than most countries and an enormous cost (19). The factors determining the huge variation in national responses have been assessed and factors such as availability of universal health care and health system capacity clearly played a role (24, 25). In addition, some governments attempted to balance protection of their economy with protecting public health; ignoring the old adage that ‘there is no wealth without health' or the old wisdoms: “The first wealth is health” (Ralph Waldo Emerson, 1860) and “It is health that is real wealth and not pieces of gold and silver.” (Mahatma Gandhi). After the first wave of the pandemic, an interesting analysis compared the timing when governments imposed national interventions (such as lockdowns) with the contemporaneous trajectory of case numbers, case fatality rates and projections of economic effects (calculated from gross domestic product (GDP) figures provided by the International Monetary Fund) and from these the authors calculated the “price of life” (26): an estimate of the economic activity that governments were prepared to lose in order to protect the life of a citizen. This varied from >$11,000,000 per life in South Korea and >$6,000,000 in New Zealand to just $87,000 in the USA and $67,000 in the UK (26). The different approaches taken by various countries will continue to present big public health challenges, some of which are yet to be addressed. In some countries, such as the UK, the government is now informing the public to “get vaccinated” and to “learn to live with the virus;” an approach that was never tried anywhere for previous epidemics of Ebola, Zika, MERS, or SARS. In countries, such as South Korea and New Zealand that successfully adopted the same rigorous public health measures to contain and eradicate SARS-CoV-2, the virus causing COVID-19, they will eventually be faced with how to deal with the resumption of global travel and the movement of people from countries that are “living” with a virus that is continuously mutating.

In the coming decades the COVID-19 pandemic will be dwarfed by even larger public health challenges as humans will have to adapt to higher temperatures (already the wet-bulb temperature beyond which humans can survive has been exceeded for brief periods in some locations), more frequent extreme whether events, major crop failures and unprecedented mass population migrations as parts of the planet become uninhabitable. According to the United Nations High Commission for Refugees, already over the last decade an estimated average of 20 million people have been forced to move home every year due to extreme weather events, such as abnormally heavy rainfall, prolonged droughts, desertification, environmental degradation, or sea-level rise and cyclones (https://www.unhcr.org/uk/climate-change-and-disasters.html). This is equivalent to the entire population of Beijing being forced to move home every year, a figure that will only get worse as the climate crisis deepens, with enormous implications for health provision. In addition, further viral pandemics seem inevitable and all of these will present big challenges to public health. The enormous cost of dealing with the COVID-19 pandemic, both economic and in mortality and morbidity, should add to the impetus to realign future government policies with much more emphasis on protecting public health.

## Author Contributions

The author confirms being the sole contributor of this work and has approved it for publication.

## Conflict of Interest

The author declares that the research was conducted in the absence of any commercial or financial relationships that could be construed as a potential conflict of interest.

## Publisher's Note

All claims expressed in this article are solely those of the authors and do not necessarily represent those of their affiliated organizations, or those of the publisher, the editors and the reviewers. Any product that may be evaluated in this article, or claim that may be made by its manufacturer, is not guaranteed or endorsed by the publisher.

## References

[1] Logette E Lorin C Favreau CPH Oshurko E Coggan JS Casalegno F. Elevated blood glucose levels as a primary risk factor for the severity of COVID-19. Front Public Health. (2021) 9:695139. 10.3389/fpubh.2021.695139 34395368 PMC8356061

[2] Zeberg H Pääbo S. The major genetic risk factor for severe COVID-19 is inherited from Neanderthals. Nature. (2020) 587:610–2. 10.1038/s41586-020-2818-3 32998156

[3] Zhou S Butler-Laporte G Nakanishi T Morrison DR Afilalo J Afilalo M. A Neanderthal OAS1 isoform protects individuals of European ancestry against COVID-19 susceptibility and severity. Nat Med. (2021) 27:659–67. 10.1038/s41591-021-01281-1 33633408

[4] Kerner G Patin E Quintana-Murci L. New insights into human immunity from ancient genomics. Curr Opin Immunol. (2021) 72:116–25. 10.1016/j.coi.2021.04.006 33992907 PMC8452260

[5] Byfield MP Murray JT Backer JM. hVps34 is a nutrient-regulated lipid kinase required for activation of p70 S6 kinase. J Biol Chem. (2005) 280:33076–82. 10.1074/jbc.M507201200 16049009

[6] Mellor P Furber LA Nyarko JN Anderson DH. Multiple roles for the p85a isoform in the regulation and function of PI3K signalling and receptor trafficking. Biochem J. (2021) 441:23–37. 10.1042/BJ20111164 22168437

[7] Islami F Goding Sauer A Miller KD Siegel RL Fedewa SA Jacobs EJ. Proportion and number of cancer cases and deaths attributable to potentially modifiable risk factors in the United States. CA Cancer J Clin. (2018) 68:31–54. 10.3322/caac.21440 29160902

[8] Holly JM Zeng L Perks CM. Epithelial cancers in the post-genomic era: should we reconsider our lifestyle? Cancer Metastasis Rev. (2013) 32:673–705. 10.1007/s10555-013-9445-5 23907184 PMC3843373

[9] Gunst J De Bruyn A Van den Berghe G. Glucose control in the ICU. Curr Opin Anaesthesiol. (2019) 32:156–62. 10.1097/ACO.0000000000000706 30817388 PMC6774765

[10] Florio G Zanella A Pesenti A. Preparedness of ICU networks for pandemics. Curr Opin Crit Care. (2021) 27:13–9. 10.1097/MCC.0000000000000792 33315637

[11] Harris G Adalja A. ICU preparedness in pandemics: lessons learned from the coronavirus disease-2019 outbreak. Curr Opin Pulm Med. (2021) 27:73–8. 10.1097/MCP.0000000000000749 33332879 PMC7924926

[12] Oakley C Pascoe C Balthazor D Bennett D Gautam N Isaac J. Assembly Line ICU: what the Long Shops taught us about managing surge capacity for COVID-19. BMJ Open Qual. (2020) 9:e001117. 10.1136/bmjoq-2020-001117 33277292 PMC7722360

[13] Taylor R Ramachandran A Yancy WS Jr Forouhi NG. Nutritional basis of type 2 diabetes remission. Br Med J. (2021) 374:n1449. 10.1136/bmj.n1449 34233884 PMC8261662

[14] Siegel KR McKeever Bullard K Ali MK Stein AD Kahn HS Mehta NK. The contribution of subsidized food commodities to total energy intake among US adults. Public Health Nutr. (2016) 19:1348–57. 10.1017/S1368980015002414 26322920 PMC10270992

[15] Franck C Grandi SM Eisenberg MJ. Agricultural subsidies and the American obesity epidemic. Am J Prev Med. (2013) 45:327–33. 10.1016/j.amepre.2013.04.010 23953360

[16] Birt CA. Food and Agriculture Policy in Europe. AIMS Public Health. (2016) 21:131–9. 10.3934/publichealth.2016.1.131 29546152 PMC5690269

[17] Center for Health Security NTI and Economist Intelligence Unit. Global Health Security Index. Washington, DC: Nuclear Threat Initiative (2019). Available online at: http://ghsindex.org/ wp-content/uploads/2020/04/2019-Global-Health-Security-Index.pdf.

[18] Dalglish S. COVID-19 gives the lie to global health expertise. Lancet. (2020) 395:1189. 10.1016/S0140-6736(20)30739-X 32222159 PMC7194526

[19] Cheng H-Y Cohen T Lin H-H. Test, trace, and isolate in the, U. K Gaps in adherence undermine effectiveness at every stage. BMJ. (2021) 372:n822. 10.1136/bmj.n822 33789886

[20] Oliu-Barton M Pradelski BSR Aghion P Artus P Kickbusch I Lazarus JV. SARS-CoV-2 elimination, not mitigation, creates best outcomes for health, the economy, and civil liberties. Lancet. (2021) 397:2234–6. 10.1016/S0140-6736(21)00978-8 33932328 PMC8081398

[21] Saurabh S Prateek S. Role of contact tracing in containing the 2014 Ebola outbreak: a review. Afr Health Sci. (2017) 17:225–36. 10.4314/ahs.v17i1.28 29026397 PMC5636234

[22] Lewis D. Why many countries failed at COVID contact-tracing - but some got it right. Nature. (2020) 588:384–7. 10.1038/d41586-020-03518-4 33318682

[23] Smith LE Potts HWW Amlôt R Fear NT Michie S Rubin GJ. Adherence to the test, trace, and isolate system in the UK: results from 37 nationally representative surveys. BMJ. (2021) 372:n608. 10.1136/bmj.n608 33789843 PMC8010268

[24] Greer SC King EJ Massard da Fonseca E Peralta-Santos A. The comparative politics of COVID-19: the need to understand government responses. Global Public Health. (2020) 15:1413–6. 10.1080/17441692.2020.1783340 32564670

[25] Chen YY Assefa Y. The heterogeneity of the COVID-19 pandemic and national responses: an explanatory mixed-methods study. BMC Public Health. (2021) 21:835. 10.1186/s12889-021-10885-8 33933062 PMC8087883

[26] Balmford B Annan JD Hargreaves JC Altoè M Bateman IJ. Cross-country comparisons of Covid-19: policy, politics and the price of life. Environ Resour Econ. (2020) 4:1–27. 10.1007/s10640-020-00466-5 32836862 PMC7400753

